# Serological screening in a large-scale municipal survey in Cascais, Portugal, during the first waves of the COVID-19 pandemic: lessons for future pandemic preparedness efforts

**DOI:** 10.3389/fpubh.2024.1326125

**Published:** 2024-02-02

**Authors:** Sofia G. Seabra, Francisco Merca, Bernardo Pereira, Ivo Fonseca, Ana Cláudia Carvalho, Vera Brito, Daniela Alves, Pieter Libin, M. Rosário O. Martins, Mafalda N. S. Miranda, Marta Pingarilho, Victor Pimentel, Ana B. Abecasis

**Affiliations:** ^1^Global Health and Tropical Medicine, GHTM, Associate Laboratory in Translation and Innovation Towards Global Health, LA-REAL, Instituto de Higiene e Medicina Tropical, IHMT, Universidade NOVA de Lisboa, UNL, Rua da Junqueira 100, Lisbon, Portugal; ^2^Artificial Intelligence Research Lab, Vrije Universiteit Brussels (VUB), Pleinlaan 2, Brussel, Belgium; ^3^Câmara Municipal de Cascais, Cascais, Portugal; ^4^Interuniversity Institute of Biostatistics and Statistical Bioinformatics, Data Science Institute, Hasselt University, Hasselt, Belgium

**Keywords:** serological survey, SARS-CoV-2, COVID-19, antibodies, RT-qPCR, sociodemographic, clinical, pandemic

## Abstract

**Background:**

Serological surveys for SARS-CoV-2 were used early in the COVID-19 pandemic to assess epidemiological scenarios. In the municipality of Cascais (Portugal), serological testing combined with a comprehensive socio-demographic, clinical and behavioral questionnaire was offered to residents between May 2020 and beginning of 2021. In this study, we analyze the factors associated with adherence to this municipal initiative, as well as the sociodemographic profile and chronic diseases clinical correlates associated to seropositivity. We aim to contribute with relevant information for future pandemic preparedness efforts.

**Methods:**

This was a cross-sectional study with non-probabilistic sampling. Citizens residing in Cascais Municipality went voluntarily to blood collection centers to participate in the serological survey. The proportion of participants, stratified by socio-demographic variables, was compared to the census proportions to identify the groups with lower levels of adherence to the survey. Univariate and multivariate logistic regression were used to identify socio-demographic, clinical and behavioral factors associated with seropositivity.

**Results:**

From May 2020 to February 2021, 19,608 participants (9.2% of the residents of Cascais) were included in the study. Based on the comparison to census data, groups with lower adherence to this survey were men, the youngest and the oldest age groups, individuals with lower levels of education and unemployed/inactive. Significant predictors of a reactive (positive) serological test were younger age, being employed or a student, and living in larger households. Individuals with chronic diseases generally showed lower seroprevalence.

**Conclusion:**

The groups with low adherence to this voluntary study, as well as the socio-economic contexts identified as more at risk of viral transmission, may be targeted in future pandemic situations. We also found that the individuals with chronic diseases, perceiving higher risk of serious illness, adopted protective behaviors that limited infection rates, revealing that health education on preventive measures was effective for these patients.

## 1 Introduction

SARS-CoV-2 (Severe acute respiratory syndrome coronavirus 2) virus was responsible for the COVID-19 pandemic, declared on the March 11th, 2020, by the World Health Organization (1), causing more than 768 million confirmed infections and over 6·9 million deaths worldwide as of June, 2023.^1^ The pandemic caused huge disruption on health systems, economy, education, and society (2). One of the public health strategies carried out early in the pandemic was the conduction of serological surveys to estimate SARS-CoV-2 seroprevalence in the general community or in target populations (3–6). Systematic reviews and meta-analyses of SARS-CoV-2 serological studies from several countries published until August 2020 found a seroprevalence ranging from 0.37 to 22.1%, with a pooled estimate of 3.4% (6), much higher than the reported cumulative number of cases (4). Chen et al. (5) and Bobrovitz et al. (3) reviewed the serological studies published until December 2020 and found pooled seroprevalences in the general population around 4.5%, with consistently higher seroprevalence in close contacts and in high-risk health-care workers, and no differences between men and women (3, 5). In northern Italy, a European region that was heavily affected in the first wave of the pandemic, the overall seroprevalence of anti-SARS-CoV-2 antibodies during March and April 2020 was 11% (7). In Spain, another considerably affected European country, the seroprevalence estimated at end of April/beginning of May 2020 was 5% (8).

In Portugal, on March 2nd, 2020, the first two cases of infection by the SARS-CoV-2 virus were confirmed and by the end of that month more than 7,000 cases were confirmed (9). The first state of emergency in Portugal was declared on March 18, 2020, with a general confinement, which included closing schools, teleworking, home confinement, closure of facilities and establishments. At that time, there was an overall reduction in population mobility of 80% (10). The period from March to the end of April 2020 corresponded to the “first wave” of the disease in Portugal, with the incidence peak having occurred on the 10th of April 2020 (1,500 new cases). The nationwide seroprevalence estimated from May to July 2020 was of 2.9% (11). In May 2020 the confinement relief plan took of (12) and, in that same month, Cascais Municipal Council was the first Portuguese municipality, and among the earliest in the world, to offer to its entire population, free of charge, the possibility of performing serological testing to detect antibodies to SARS-CoV-2, combined with a questionnaire. The Municipality of Cascais, composed of four parishes (“freguesias”), with Cascais as the municipal seat, is in the Lisbon Metropolitan Area (Portugal) and had 214,124 inhabitants in 2021.^2^ The serological survey was carried out until February 2021 (13) and included two additional epidemic waves that started in October and December 2020, respectively, with higher incidence than the first wave. In February–March 2021, the estimated nationwide seroprevalence was estimated to be 15.5% (11).

We aimed to analyze the adherence to the serological survey, as well as its results to identify the challenges in this type of regional study. Our objectives were: (1) to identify the population groups with lower adherence to the survey; (2) to characterize the socio-demographic profile of the most at-risk groups; (3) to understand the impact of chronic diseases in the exposure to the virus.

## 2 Materials and methods

**Figure 1:**
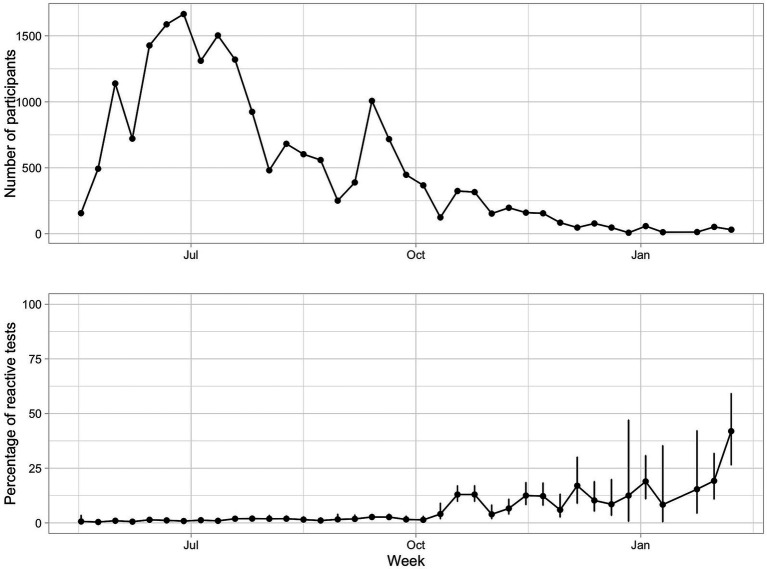
Number of participants answering the questionnaires and seroprevalence (%) per week, between May and October 2020. The bars are 95% confidence intervals.

This is a cross-sectional study with non-probabilistic sampling. It is based on secondary data collected in a SARS-CoV-2 serological survey to citizens residing in the Portuguese Municipality of Cascais who voluntarily decided to participate (non-probabilistic sampling). Participants went to blood collection centers to carry out the serological test and to fill out a questionnaire. The participants gave their informed consent to their anonymized data being used for statistical analysis purposes. The study ran from May 21st 2020 to February 12th 2021. Two distinct pandemic phases were recorded and were analyzed independently (Figure 1). One ran from May 2020 to October 2020, during the phases of confinement relief in the first wave of the pandemic, when a large number of people participated in the study. The second period ran from October 2020 to February 2021, during the second wave, when fewer people participated. In the last period of this second wave, vaccination started (on 27th December 2020). At the end of February 2021, 6% of the population had had at least one dose of vaccination (13).

The serological test used for detection of antibodies (including IgG) against SARS-CoV-2 was the Elecsys Anti-SARS-CoV-2 from Roche Diagnostics, Switzerland. This test has a sensitivity after 14 days of 99.5% (95% CI, 97.0 to 100.0%), assessed using clinical samples from patients who had previously tested positive for SARS-CoV-2 by PCR, and a specificity of 99.8% (95% CI, 99.69 to 99.88%), assessed using clinical samples collected before December 2019 (14). This test does not detect antibodies elicited by vaccination.

The variables under analysis were of the following types: sociodemographic, chronic diseases background, COVID-19 background and medical support and absenteeism (Supplementary Table). Based on the professional activity reported by participants who were employed, we created another variable: health professional (yes/no). Questions only added to the questionnaire from July 2020 onwards were: country of birth; size of household; occurrence of chronic kidney disease. We report the proportion of missing data for each variable. All variables were categorical, except age and size of household, which were converted to categorical (Table 1).

**Table 1:** Socio-demographic characterization of the respondents to the COVID-19 serological survey of the Municipality of Cascais from May 2020 to February 2021.

Variable	Category	N	%
Gender	Female	11,678	60.3
	Male	7,685	39.7
Age group	0–9	341	1.8
	10–19	1,528	7.9
	20–29	1813	9.4
	30–39	2,541	13.2
	40–49	4,179	21.7
	50–59	3,378	17.5
	60–69	2,974	15.4
	70+	2,507	13.0
Level of education	Basic1	1,366	7.1
	Basic2	1,062	5.5
	Basic3	2,116	11.0
	Medium	2,270	11.8
	Secondary	4,043	21.0
	Superior	7,633	39.7
	No scholarity	757	3.9
Employment status	Retired	3,901	20.3
	Employed	10,484	54.6
	Student	2,336	12.2
	Unemployed	1767	9.2
	Other	702	3.7
Health profession	No	9,384	92.2
	Yes	796	7.8
Country of birth	Other countries	1,453	24.2
	Portugal	4,548	75.8
Residence locality	Alcabideche	2,692	14.8
	Carcavelos / Parede	3,910	21.5
	Cascais / Estoril	7,058	38.8
	São Domingos de Rana	4,509	24.8
Size of household	1	1,122	19.1
	2–3	2,905	49.4
	4–5	1705	29.0
	5+	150	2.6

We analyzed the representativeness of the different categories of respondents by comparing proportions of the categories in the survey with the socio-demographic census description of the Cascais municipality (census of 2021) available in PORDATA (15), using the chi-square test.

The rate of seropositives for antibodies against SARS-CoV-2 was stratified by socio-demographic and clinical variables. We additionally used the approach by Larremore et al. (16) to obtain the posterior distribution of seroprevalence from a Bayesian model that incorporated uncertainty from test sensitivity and specificity and seroprevalence heterogeneity across subpopulations. Association between seropositivity and each categorical variable was analyzed with odds ratio, chi-square test (with continuity correction for tables 2×2) or Fisher exact test (when expected frequencies were lower than 5), and Wald test from simple logistic regression. Multivariable logistic regression using socio-demographic variables as predictors and the result of the serological test (reactive or non-reactive) as outcome, was carried out using R package MASS. The trend of seropositivity along time (in weeks) was analyzed using logistic regression.

The significance level considered was 5%. Statistical analyses were performed in R version 4.1.0 (R Core Team, 2021).^3^

We followed the STROBE cross sectional reporting guidelines (17).

## 3 Results

### 3.1 Characterization of the sample

A total of 21,373 questionnaires with serological test result were obtained from May 21^st^ 2020 to February 12th 2021, corresponding to a response rate of 9.98% of the resident population of Cascais (214,158 residents in Cascais in 2021, according to the national census). The date of the serological test was missing for 1765 questionnaires, and these were excluded, leaving a total of 19,608 questionnaires for analysis. 60.3% of the participants were women, 39.7% men, 23 participants answered “Other” and 71 participants answered “Do not know/does not answer.” The median age of the participants was 48 years old (interquartile range - IQR 27). 39.7% had higher education and other 21% completed high school. 54.6% were employed. From the four localities (parishes, i.e., “Freguesias”) of Cascais municipality, “Cascais / Estoril” had a higher proportion of participants (38.8%; Table 1). From the 6,001 that responded to the question about country of birth, 24.2% were born outside Portugal (57 countries), with a predominance of Brazil (44.6%), followed by Angola (15.1%), Mozambique (10.7%), France (3.4%) and the United Kingdom (2.3%).

Comparing with the proportions in the general population of Cascais (15), some categories had lower representation in the questionnaires: men (*p* < 0.0001), the youngest and the oldest age groups (*p* < 0.0001), lower levels of education (*p* < 0.0001), unemployed and other inactives (*p* <0.0001). Immigrants had higher representation in this survey than the proportion found in the census (*p* < 0.0001; Supplementary Figure 1).

Over 88% of the questionnaires were collected in the first period (17,309 questionnaires from May 21st to October 1st 2020), with a mean of 869 (ranging from 156 to 1,665) questionnaires per week. The remaining 2,299 questionnaires were collected from October 2020 to February 2021 (mean of 124 per week, ranging from 8 to 367; Figure 1).

### 3.2 Seropositivity for SARS-CoV-2 and time trend

In the first period (May 21st to October 1st 2020), from the 17,309 surveys analyzed, 241 had a reactive serological test, that is, an overall proportion of seropositives of 1.40% (95% confidence interval C.I.: 1.23–1.59%). Considering the known sensitivity and specificity of the serological test, the Bayesian posterior estimate of seroprevalence was 1.21% (95% credible interval: 1.07–1.37%). In the second period (October 2020 to February 2021), from the 2,299 surveys analyzed, 217 had a reactive serological test, that is, the estimated seroprevalence was 9.44% (95% C.I.: 8.31–10.70%). Bayesian posterior estimate was 9.35% (95% credible interval: 8.35–10.38%).

Across the 38 weeks of the study, the percentage of reactive cases per week showed an increasing trend (value of *p* < 2e-16), ranging from 0.4% (95% C.I.: 0.11–1.47%) in the second week to 41.9% (95% C.I.: 26.4–59.2%) in the last week (Figure 1). The two periods of the study demonstrate different characteristics: in the first period there was a large number of questionnaires per week (people were highly motivated to participate at the start of the pandemic) and the proportion of seropositives was still low (Figure 1), while in the second period the number of questionnaires per week was much lower and the proportion of seropositives were much higher, as expected as the pandemic progressed. Due to these marked differences, data analyses were done independently for each period.

### 3.3 Seropositivity for SARS-CoV-2 according to socio-demographic factors

**Figure 2:**
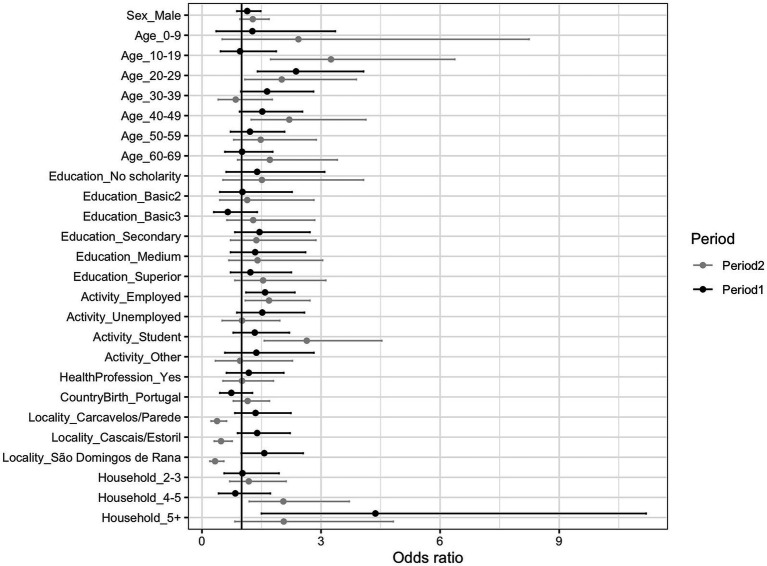
Odds ratio (and 95% confidence intervals) of a reactive serological test outcome for each sociodemographic category, at each period of the study. Reference categories are indicated in Supplementary Table S1.

Considering the first period, the odds of having a reactive serological test was not significantly different between sexes nor levels of education (Figure 2; Supplementary Table S1), but it was significantly higher in the 20–29 age group relative to the 70+ reference group (odds ratio OR: 2.37, 95% C.I.: 1.40–1.87, *p* = 0.001), and in employed people when compared to retired people (OR: 1.59, 95% C.I.: 1.11–2.34, *p* = 0.014). Health professionals had similar seroprevalence to the general population. People living in one of the localities of the municipality, “São Domingos de Rana,” had higher odds of a reactive test when compared to the locality “Alcabideche.” People within large households (more than 5 people) showed significantly higher odds of having a reactive serological test than people living in single households (OR: 4.37, 95% C.I.: 1.50–11.19%, *p* = 0.003). Participants belonging to these large households (> 5) were mainly employed (60%) or students (21%), while only 5% of people reported living in such large households were retired (this group represents 21% in the overall participants). 37% of participants living in large households were from Cascais/Estoril (similarly to the overall proportion of inhabitants of this locality in the sample, which was 39%), but we found an over-representation of participants from “São Domingos de Rana” in these large households (32% against 24% overall proportion of inhabitants of this locality in the sample) and a sub-representation of large households in “Alcabideche” (10% against 16% overall proportion of inhabitants of this locality).

For those 4,410 participants who reported country of birth in the first period of the study, the proportion of seropositives did not differ significantly between those born in Portugal and those born abroad (Supplementary Table S2). Within those born abroad, when stratifying by age group, education level and professional level, some groups were identified as having high seroprevalence, for example, the 20–29 age group, 1^st^ and 2^nd^ basic education level and unemployed, resident at “São Domingos de Rana,” and living in large households, although the small sample size contribute to the large CI (Supplementary Figure 2).

When controlling for all the socio-demographic variables (multivariate logistic regression analysis), higher odds of a reactive serological test were found for people living in households with more than 5 people compared to the single households (adjusted *p* = 0.0181) (Supplementary Table S3).

In the second period, the patterns were similar as described for the first period for most variables, but with higher seroprevalence values (Supplementary Table S2). People aged 10–29 and 40–49 showed higher odds of a reactive test than the 70+ reference group, as well as employed people and students than retired people. Contrarily to the first period, in the second period seroprevalence in “Alcabideche” was higher than in the other localities. Households with more members also had higher seroprevalence, although only significant in the un-adjusted logistic regression (Supplementary Tables S2, S3). Contrarily to the first period, in the second period there was an over-representation of people from “Alcabideche” in the group of people living in large households (> 5 persons) (23% compared to 8% of inhabitants in “Alcabideche” in the overall sample in the second period) and a sub-representation of “São Domingos de Rana” (8.9% compared to 28% in the overall population).

### 3.4 Seropositivity for SARS-CoV-2 and chronic diseases

In the first period, 32.7% of the participants reported having at least one chronic disease, while 8.5% reported at least two chronic diseases. For each of the chronic diseases, seroprevalence was generally lower in the chronically ill, but OR was not significant (Supplementary Table S4). In the second period, the chronically-ill also showed lower seroprevalence estimates than the non-chronically ill for all diseases, with significant differences (*p* < 0.05) found for Diabetes and Autoimmune disease (Supplementary Table S5).

### 3.5 Seropositivity for SARS-CoV-2 according to reported COVID-19 background

**Figure 3:**
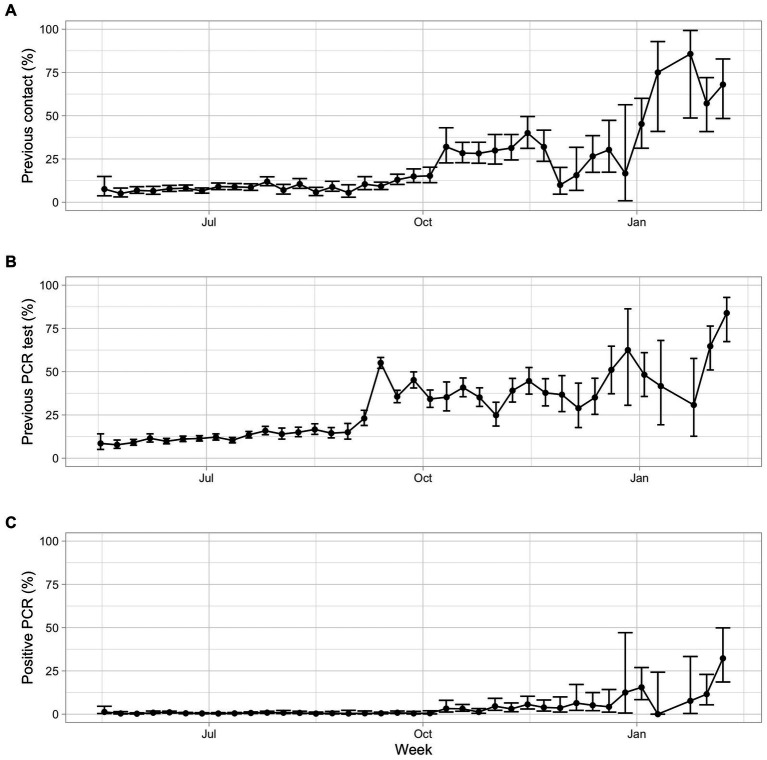
Proportion of participants, per week: **(A)** reporting previous contact with suspected or confirmed case; **(B)** reporting having done a previous RT-qPCR test; **(C)** reporting having had a positive RT-qPCR test (from the total participants).

In both periods, the proportion of seropositives was significantly higher in those reporting a previous risk contact (Supplementary Tables S4, S5). An increasing trend was observed in the proportion of reported contacts along the weeks, with a steep increase in the month of October 2020 and another in January 2021 (Figure 3A).

For the total study, 3,572 participants (18.9% of the respondents) reported having previously performed a RT-qPCR test to detect the virus. During the period of study, there was an increasing trend in this proportion, from an estimate lower than 10% in the first weeks to higher than 50% in the last weeks (Figure 3B). 189 participants (0.96% of the total respondents and 5.6% of those that did a RT-qPCR test) reported having had a positive RT-qPCR test, therefore who reported having had a confirmed prior infection. Considering the total participants in each week of the study, the proportion of positive RT-qPCR test was very low in the first period (overall proportion of 0.04%) and it was much higher in the second period (overall proportion of 10.5%, from around 5% in the weeks of October to December, reaching over 10% of the total questionnaires in the weeks of January and over 30% in the first week of February) (Figure 3C). From the respondents who reported having had a positive RT-qPCR test (189 in total), 75.66% of them had a reactive serological test, which means that, in about a quarter of those infected, no anti-SARS-CoV-2 antibodies were subsequently detected. From these 46 participants, 67% were female, and 8.9% were children younger than 10 years old, proportions higher than the general proportion of participants in these groups (60.3 and 1.8%, respectively; Table 1).

From the 252 participants that had a reactive serological test and that reported a previous RT-qPCR result, 43% reported a negative RT-qPCR test result. This proportion was the same in both periods (53 out of 123 participants in the first period and 56 out of 129 in the second period), which is visualized in Supplementary Figure S3.

### 3.6 Demand for medical support and absenteeism

In the first period, 1,451 persons (9.8% of the respondents) reported having looked for medical support because of the symptoms, 143 (1% of the respondents) reported having been hospitalized and 17 (0.4% of the respondents) reported having been in intensive care. The reported absenteeism was of 532 persons (3.7% of respondents). In the second period, the proportions were similar, except for the reported absenteeism that was much larger (9.3% of respondents) (Supplementary Tables S4, S5). In both periods, seropositivity was associated with demand for medical support and with absenteeism (Supplementary Tables S4, S5).

## 4 Discussion

The serological survey carried out by the Municipality of Cascais allowed the collection of extensive socio-demographic and clinical (chronic diseases) data from a large number of participants and to associate it with the results of the serological screening to detect antibodies against the SARS-CoV-2 virus. In this study, we analyzed the results from May 2020 to February 2021, in two distinct periods, the first corresponding to the relief stages after the first wave of the COVID-19 pandemic, and the second during the second and third waves, from October 2020 to February 2021.

This study was based on a non-random volunteer sample, which covered almost 10% of the residents of Cascais. The participants did not pay for the test, and they were informed of the test result. The success of this initiative, as assessed by the coverage of the population reached, shows the importance of this type of public health intervention and its ability to mobilize local populations in times of pandemic. One major pitfall of this study was the fact that questionnaires were delivered in paper. This fact implied that around 20,000 questionnaires had to be inserted manually in a database of the study, which significantly delayed the analysis and dissemination of the results.

Compared to census data, there was lower representativeness of men, the youngest and the oldest age groups, lower levels of education and unemployed/inactives. This information is relevant to design strategies to include underrepresented groups in future serological surveys offered to the population. The gender difference that we found, with less than 40% of participants being men, may be explained by a general underutilization of preventive health care services by men (e.g., (18, 19)). Moreover, earlier work also demonstrated that men exhibit less adherence to public health recommendations (20), which could be another contributing factor.

The proportion of seropositives for SARS-CoV-2 in the population of Cascais from May to September 2020 was of 1.40% (95% C.I.: 1.23–1.59%), lower than the 2.9% obtained in May–July at national level (21), which may be related to lower risk exposure factors in Cascais compared to the national situation. However, it should be noted that the surveys have different sampling methods, thus the comparison is only indicative. The second phase of the national survey was carried out in February and March 2021, revealing an overall estimated seroprevalence of 15.5% (IC 95: 14.6–16.5%) at that time (22, 23). In the second period of our study in Cascais (October 2020 to February 2021), the overall proportion of seropositives was 9.44% (95% C.I.: 8.31–10.70%), reaching values of more than 10% in January – February 2021, also lower than in the national survey.

No significant difference was found in the proportion of seropositives between sexes, as in several other studies (6, 8, 24, 25). However, the age group 20–29 years old presented significantly higher odds of being seropositive than the older age groups in both periods. This is a group that showed increased risk of infection in subsequent stages of the pandemic due to higher social interaction (26, 27). Older age groups had lower odds, which may reflect the adoption of effective protective behaviors from infection in these age groups since early in the pandemic. However, this study does not include the older adults living in care homes that had higher risk of infection throughout the pandemic (8, 28) and is biased toward the people that were able to visit the testing centers.

Higher odds of seropositivity were found in employed people and students when compared to retired people but this association was not maintained when controlling for other factors. Interestingly and unexpectedly, the workers in health professions did not have higher odds of seropositivity than the general population. While unexpected, this result is coherent with what was found previously in hospital settings, even when discriminating the activities with direct patient contact (29, 30). This suggests that protective measures are generally efficient and that, in health workers, the infection is mainly acquired through community or household instead of through patient contact. However, in a study by Grant et al. (31), seropositivity was found to be higher in health workers with direct patient contact than in those without. Other professional activities with high risk of exposure also deserve examination.

There was no significant difference between SARS-CoV-2 seroprevalence between immigrants and those born in Portugal, although immigrants belonging to the 20–29 age group, with lower levels of education level, unemployed and living in larger households showed higher proportion of seropositives (but with large C.I.). The health inequalities that affect migrant populations are already well known and also reflected in the greater impact of COVID-19 on these populations (3, 32, 33). However, it should be noted that these inequalities are often related to socio-economic status and not to the country of birth. In Cascais, one of the wealthiest municipalities in Portugal, this is particularly striking. Factors for increased risk should be considered and questionnaires designed to address migrants’ specificities and dimensions of vulnerability should be added in future sero-surveys, e.g., professional occupations requiring personal contact, commuting to work in public transportation, access to health care, etc.

The results indicate that factors related to housing are also associated with higher risk of viral transmission. Herein, larger households were associated with higher seropositivity, similarly to what was found in other studies (24, 28, 34, 35). Household transmission of SARS-CoV-2 has been identified early in the pandemic and quarantine of infected individuals within household has been one of the suggested measures to prevent transmission (36, 37). Crowded houses preclude these isolation measures. On the other hand, when comparing the results of the two periods, we found higher seropositivity in two different localities within the municipality. This could be a sampling effect due to differential representation of, for example, people living in large households coming from these two localities in the two periods.

According to the Survey on Living Conditions and Income 2020–2021 (38), in the Lisbon Metropolitan Area, where the Municipality of Cascais is located, the proportion of the population aged 16 and over with a chronic illness or long-term health problem was 41.4 per cent in 2020 and 42.6 per cent in 2022, higher percentages than those reported in our study (around 30%). Participants with chronic illnesses showed generally lower seroprevalence, similarly to what was found in those with chronic diseases in the United States (39). In general, chronically ill patients may have adopted protective measures to avoid infection since the higher risk of severe outcomes of COVID-19 in the chronically ill was widely announced since the start of the pandemic. However, as we mentioned before, our sampling, based on voluntary displacement to testing centers, limited the participation of citizens with low mobility and chronically ill individuals with higher levels of morbidity may not be well represented in the sample.

Interestingly, this study also allowed to quantify the discrepancy between the RT-qPCR outcome (reported by respondents) and the serological results. From those participants that had a previous COVID-19 infection, as indicated by serological testing, and reported to have performed a previous RT-qPCR test, 43% reported a negative RT-qPCR result. We note that we lack information about the timing of the infection and that of the PCR test, which may explain this discrepancy. The specificity of the serological test may also be lower than expected. About 75% of the participants who reported to have had a confirmed prior infection (positive RT-qPCR test) were reactive in the serological test (i.e., they had antibodies for SARS-CoV-2), which leaves 25% of confirmed infected individuals with no anti-SARS-CoV-2 antibodies subsequently detected. These corresponded to 46 individuals that included a higher proportion of females and young children compared to the proportion of these groups in the sample, but since the number of individuals is low, we could not verify the statistical significance for these trends. One potential contributing factor to the lack of serological detection might be a reduced sensitivity of the serological test, however this would need to be evaluated through a targeted study. Also, the duration of the immune response to SARS-CoV-2 infection is still poorly understood and is not only based on antibodies but also on cellular immunity (40). In this context, it is important to highlight that the serological tests done here only assess previous exposure and not immunity, as no neutralizing antibodies were measured. The initiation of the vaccination campaign started in December 2020, with a primary focus on prioritizing specific demographic groups for vaccination. At the end of this study the coverage of vaccination in the population was still very low (2.02% of the Portuguese population by 15th of February 2021) and, as previously mentioned, the serological test used in this study does not detect antibodies elicited by vaccination.

Across the study period, there was an increase in the proportion of participants that reported that they had performed a RT-qPCR test previously. In the period from May to July 2020, the proportion of persons that did a RT-qPCR test was around 10%, close to the 8.8% reported in the national survey (21). In September 2020, from 2,353 respondents, 43% had done previously a RT-qPCR test. This may reflect, not only an increase in the proportion of the population with previous RT-qPCR testing, but also a greater increase in serological testing by people that were generally more exposed. On the other hand, in September 2020 there was an increase in the proportion of participants reporting previous contact with a confirmed or suspected case, after increased public health efforts of contact tracing being put in place from July 2020 onwards (9, 41). Contact tracing was an important tool for the public health mitigation strategy in the countries where it was feasible (42).

In the first period, 0.59% of the total participants reported having had a RT-qPCR positive test. This is lower than found in the first national serological survey [0.8% (21)] and also in the national accumulated incidence value at the beginning of October 2020 [0.7% (43)]. This could be due to a lower risk of exposure in the population of Cascais, or likely in some segments of the population that were able to adopt more effective preventive measures, in comparison to other municipalities.

With this study we identified several aspects that are important to consider in future serological surveys: (1) the willingness to participate was high but some particular groups were underrepresented and should be explicitly targeted in future initiatives, either with targeted campaigns or by offering the survey closer to the target groups; (2) the option of using digital questionnaires should be considered, in order to guarantee a quicker and less error-prone data processing, as well as for the generation of fast evidence to support decision making, but this option might imply that digitally excluded persons will not participate; (3) in order to identify vulnerabilities that may be related to higher risks of exposure, it is important to include in-depth questions about socio-economic conditions, including commuting and housing, as well as about professional/school-related and leisure activities involving personal contact.

The municipality of Cascais faces large disparities in income and housing conditions. Public health policies should take these conditions into account to overcome socioeconomic and health inequalities. While we and others have previously proposed the use of mass testing to control the epidemic, herein we show that serological surveys coupled with socioeconomic questionnaires provide very important information during epidemics to understand determinants of transmission and of exposure to the virus (44). This should be considered for future pandemic preparedness efforts. In this context, municipalities are well positioned to play an active role in the implementation of these local initiatives to meet national public health goals.

## 5 Conclusion

This serological survey, combined with a comprehensive questionnaire, carried out in the municipality of Cascais, allowed (1) to identify the population groups that are less able (or willing) to participate in this type of surveys, which could be specifically targeted in future studies, namely men, younger and older age groups, unemployed and incapacitated or other inactive persons; (2) to identify the main risk factors and specific population groups more at risk of infection, namely younger immigrants, employed individuals or students, people living in larger households or in particular localities. Overall, this study will help to guide more effective preventive measures to minimize the impact of future pandemics.

## Data availability statement

The raw data supporting the conclusions of this article will be made available by the authors, without undue reservation.

## Ethics statement

Ethical approval was not required for the study involving human samples in accordance with the local legislation and institutional requirements because this study used secondary data obtained by the Municipality of Cascais during the serological survey. The municipalities in Portugal have autonomy to carry out public health initiatives without the need for an ethics committee approval. Written informed consent for participation in this study was provided by the participants or their legal guardians/next of kin.

## Author contributions

SGS: Data curation, Formal analysis, Investigation, Methodology, Supervision, Validation, Visualization, Writing – original draft, Writing – review & editing. FM: Data curation, Formal analysis, Validation, Visualization, Writing – original draft, Writing – review & editing. BP: Formal analysis, Writing – review & editing. IF: Formal analysis, Writing – review & editing. AC: Project administration, Writing – review & editing. VB: Project administration, Writing – review & editing. DA: Data curation, Writing – review & editing. PL: Writing – review & editing. MOM: Conceptualization, Methodology, Writing – review & editing. MNM: Data curation, Writing – review & editing. MP: Data curation, Writing – review & editing. VP: Data curation, Writing – review & editing. AA: Conceptualization, Methodology, Supervision, Project administration, Resources, Validation, Writing – review & editing.

## Footnotes

^1^https://covid19.who.int/

^2^https://www.pordata.pt/censos/quadro-resumo-municipios-e-regioes/cascais-574

^3^ R scripts for analyses and plotting are available at: https://github.com/seabrasg/serological_survey
