# Supplementary material for: Serological screening in a large-scale municipal survey in Cascais, Portugal, during the first waves of the COVID-19 pandemic: lessons for future pandemic preparedness efforts
Source: Front Public Health. 2024 Feb 2;12:1326125. doi: 10.3389/fpubh.2024.1326125 (PMC10869482; doi:10.3389/fpubh.2024.1326125)
Supplement: Supplementary file 1 [file Data_Sheet_1.pdf]

Supplementary Material

Serological screening in a large-scale municipal survey in Cascais, Portugal, during the first waves of the COVID-19 pandemic: lessons for future pandemic preparedness efforts

Sofia G. Seabra[1†], Francisco Merca[1,3†], Bernardo Pereira[1], Ivo Fonseca[1], Ana Cláudia Carvalho[2], Vera Brito[2], Daniela Alves[1], Pieter Libin[3], M. Rosário O. Martins[1], Mafalda Miranda[1], Marta Pingarilho[1], Victor Pimentel[1], Ana B. Abecasis[1*]

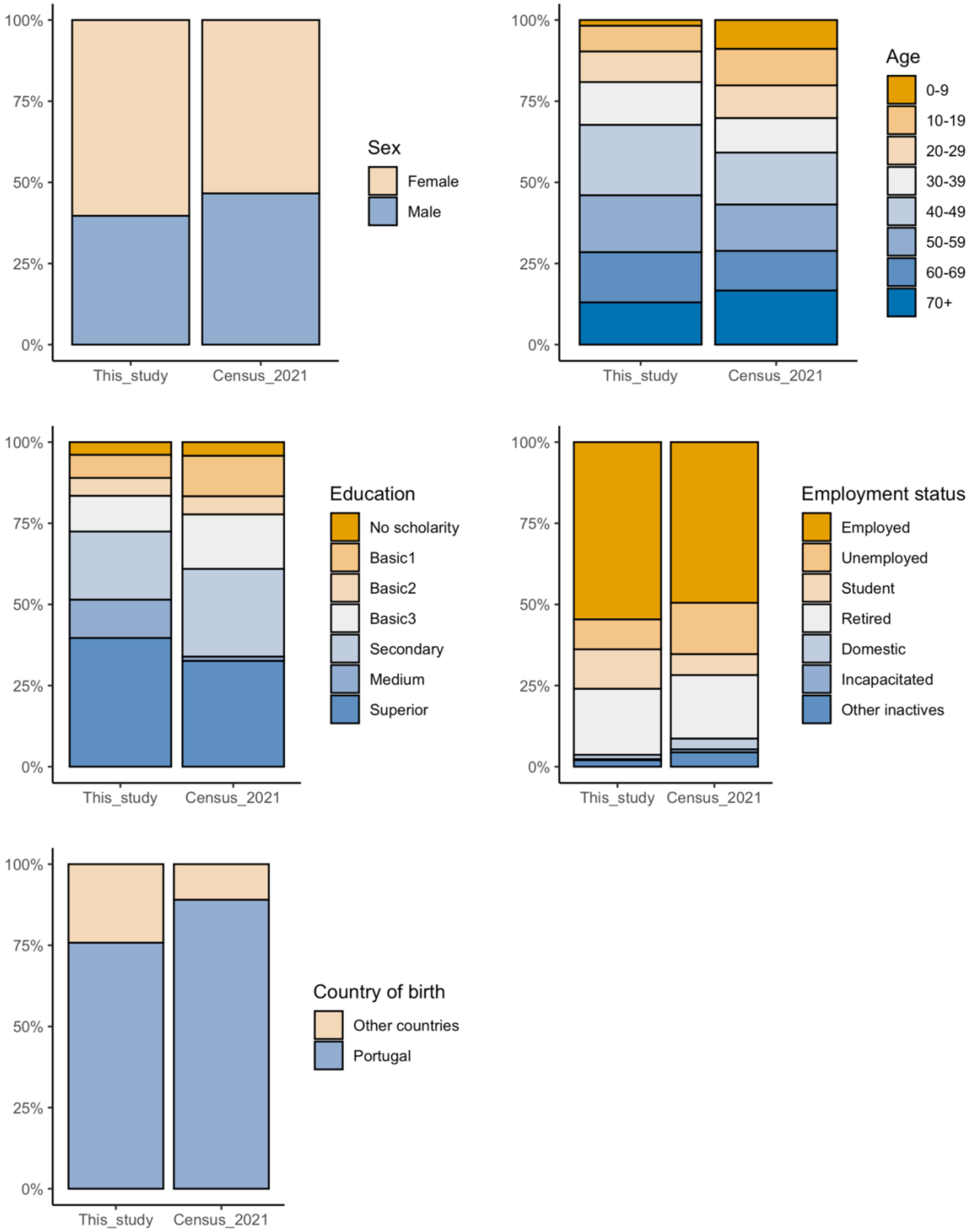

Supplementary Figure S1 –Proportions of each category of the sociodemographic variables in this study and those found in the Census 2021 for Cascais Municipality.

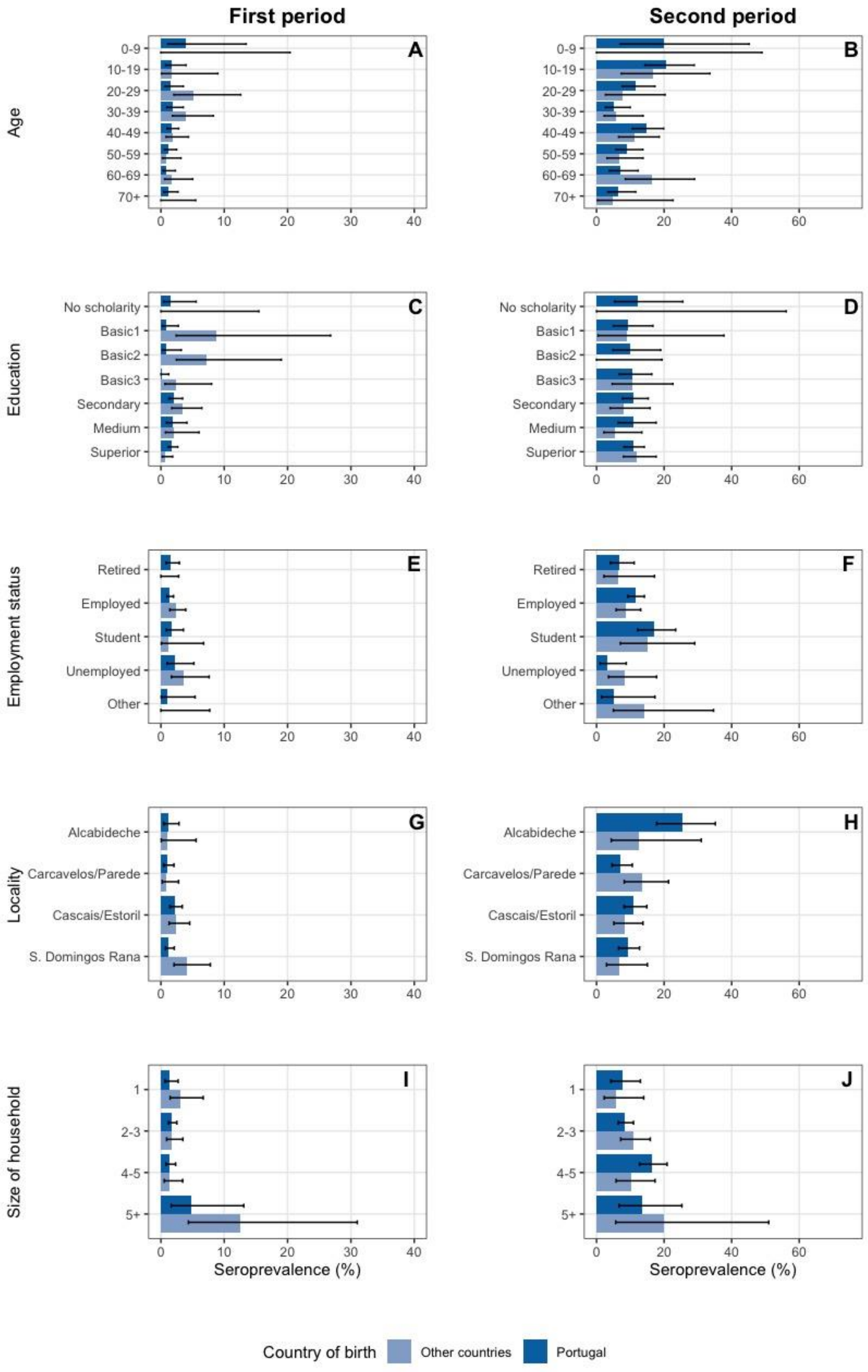

Supplementary Figure S2 – Percentage (and 95% confidence intervals) of reactive SARS-CoV-2 serological tests for each category of each variable at each period of the study and for people born in Portugal (dark blue) or in other countries (light blue). Graphs on the left refer to the first period of the study and on the right to the second period.

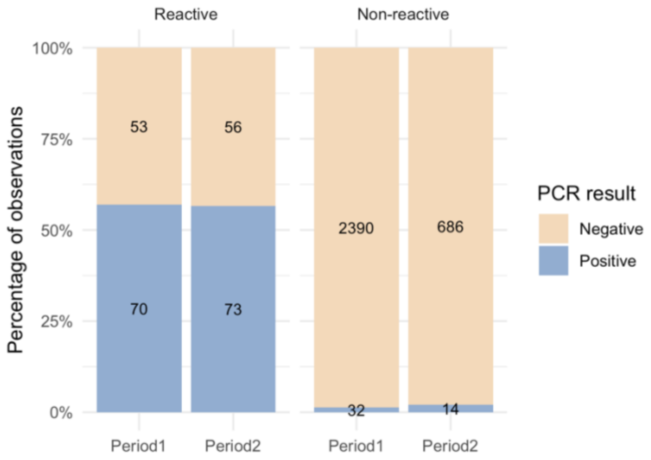

Supplementary Figure S3 – Stacked barplots of the percentage of participants with Positive or Negative PCR tests for each category of serological test result (Reactive or Non-reactive) for each period of the study.

Supplementary Table S1 - Proportion of seropositives for SARS-CoV-2 stratified by socio-demographic variables in the first period of the study. N - sample size for each category; % - proportion of each category within each variable; N sero+ - number of seropositives for SARS-CoV-2; Proportion of seropositives - in percentage and with 95% confidence interval. Association between seropositivity and each categorical variable was analysed with odds ratio, Chi-Square(C-S) test (with continuity correction for tables 2x2) or Fisher (F) exact test (‡), and Wald test from simple logistic regression. Bold values are p-values < 0.05. The numbers and percentage of missing data (NA) is given for each variable but were excluded for the calculation of proportion of each category.

First period of the study								
**Variable**	**Category**	**N**	**%**	**N sero+**	**Proportion of seropositives (95% CI) (%)**	***Odds ratio (95% CI)***	**p-value C-S or F(‡) test**	**p-value Wald test**
**Gender**	Female	10386	60.7	137	1.32 (1.12-1.56)	*ref*		
	Male	6714	39.3	101	1.50 (1.24-1.82)	*1.14 (0.88-1.48)*	0.346	0.313
	NA	*209*	*1.2*					
**Age group**	0-9	317	1.9	4	1.26 (0.49-3.20)	*1.27 (0.36 - 3.36)*	0.698	0.699
	10-19	1335	7.8	13	0.97 (0.57-1.66)	*0.96 (0.47 - 1.87)*	0.883	0.883
	20-29	1502	8.8	36	2.40 (1.74-3.30)	*2.37 (1.40 - 4.07)*	**0.001**	**0.001**
	30-39	2214	13	37	1.67 (1.21-2.29)	*1.64 (0.98 - 2.81)*	0.061	0.064
	40-49	3724	21.9	58	1.56 (1.21-2.01)	*1.52 (0.95 - 2.53)*	0.085	0.087
	50-59	2981	17.5	37	1.24 (0.90-1.71)	*1.21 (0.72 - 2.08)*	0.467	0.467
	60-69	2700	15.9	28	1.04 (0.72-1.49)	*1.01 (0.58 - 1.78)*	0.965	0.965
	70+	2245	13.2	23	1.02 (0.68-1.53)	*ref*		
	NA	*291*	*1.7*					
**Level of education**	Basic1	1206	7.1	14	1.16 (0.69-1.94)	*ref*	0.170	
	Basic2	933	5.5	11	1.18 (0.66-2.10)	*1.02 (0.45-2.27)*	0.969	0.969
	Basic3	1843	10.8	14	0.76 (0.45-1.27)	*0.65 (0.30-1.39)*	0.256	0.260
	Medium	1994	11.7	31	1.55 (1.10-2.20)	*1.34 (0.72-2.61)*	0.359	0.361
	Secondary	3548	2.9	60	1.69 (1.32-2.17)	*1.45 (0.83-2.72)*	0.199	0.201
	Superior	6788	39.9	97	1.43 (1.17-1.74)	*1.22 (0.72-2.25)*	0.463	0.464
	No scholarity	687	4	11	1.60 (0.90-2.84)	*1.39 (0.61-3.09)*	0.420	0.422
	NA	*310*	*1.8*					
**Employment status**	Retired	3513	20.7	35	1.00 (0.72-1.38)	*ref*		
	Employed	9251	54.6	146	1.58 (1.34-1.85)	1.59 (1.11 - 2.34)	**0.013**	**0.014**
	Student	2047	12.1	27	1.32 (0.91-1.91)	1.33 (0.79 - 2.20)	0.269	0.271
	Unemployed	1526	9	23	1.51 (1.01-2.25)	1.52 (0.88 - 2.58)	0.118	0.121
	Other	599	3.5	8	1.34 (0.68-2.61)	*1.37 (0.58 – 2.82)*	0.451	0.452
	NA	*373*	*2.2*					
**Health profession**	No	8316	92.6	129	1.55 (1.31-1.84)	*ref*		
	Yes	664	7.4	12	1.81 (1.04-3.13)	*1.18 (0.62-2.06)*	0.728	0.610
**Country of birth**	Other countries	1039	23.6	21	2.02 (1.33-3.07)	*ref*		
	Portugal	3371	76.4	51	1.51 (1.15-1.98)	*0.74 (0.45-1.27)*	0.322	0.258
	NA	*12899*	*74.5*					
**Residence locality**	Alcabideche	2517	15.6	25	0.99 (0.67-1.46)	*ref*		
	Carcavelos / Parede	3367	20.9	45	1.33 (1.00-1.78)	*1.35 (0.83-2.24)*	0.230	0.211
	Cascais / Estoril	6297	39.1	87	1.38 (1.12-1.70)	*1.39 (0.90-2.22)*	0.141	0.091
	São Domingos de Rana	3925	24.4	61	1.55 (1.21-1.99)	*1.57 (0.99-2.55)*	0.056	**0.048**
	NA	*1203*	*7.0*					
**Size of household**	1	900	20.6	15	1.67 (1.01-2.73)	*ref*		
	2-3	2105	48.2	36	1.71 (1.24-2.36)	*1.02 (0.56-1.94)*	0.933	0.933
	4-5	1274	29.2	18	1.41 (0.90-2.22)	*0.84 (0.42-1.72)*	0.634	0.634
	5+	88	2	6	6.82 (3.16-14.09)	*4.37 (1.50-11.19)*	**0.001**	**0.003**
	NA	*12942*	*74.8*					

Supplementary Table S2 - Proportion of seropositives for SARS-CoV-2 stratified by socio-demographic variables in the second period of the study. N - sample size for each category; % - proportion of each category within each variable; N sero+ - number of seropositives for SARS-CoV-2; Proportion of seropositives - in percentage and with 95% confidence interval. Association between seropositivity and each categorical variable was analysed with odds ratio, Chi-Square(C-S) test (with continuity correction for tables 2x2) or Fisher (F) exact test (‡), and Wald test from simple logistic regression. Bold values are p-values < 0.05. The numbers and percentage of missing data (NA) is given for each variable but were excluded for the calculation of proportion of each category.

Second period of the study

**Variable**	**Category**	**N**	**%**	**N sero+**	**Proportion of seropositives (95% CI) (%)**	***Odds ratio (95% CI)***	**p-value C-S or F test**	**p-value Wald test**
**Gender**	Female	1292	57.09	111	8.59 (7.18 -10.24)	*ref*		
	Male	971	42.91	104	10.71 (8.92 - 12.81)	*1.28 (0.96 -1.69)*	0.0888	*0.0894*
	NA	*36*	*1.6*					
**Age group**	0-9	24	1.07	3	12.5 (4.34 - 31.00)	*2.43 (0.51 - 8.24)*	0.191	0.203
	10-19	193	8.60	32	16.58 (12.0 - 22.47)	*3.25 (1.73 - 6.37)*	**0.00017**	**0.000312**
	20-29	311	13.87	34	10.93 (7.93 -14.89)	*2.01 (1.08 - 3.89)*	**0.0264**	**0.0289**
	30-39	327	14.58	16	4.89 (3.03 - 7.8)	*0.85 (0.41 - 1.77)*	0.653	0.653
	40-49	455	20.29	54	11.87 (9.21 - 15.16)	*2.20 (1.24 - 4.13)*	**0.00723**	**0.00855**
	50-59	397	17.70	33	8.31 (5.98 - 11.44)	*1.48 (0.80 - 2.88)*	0.211	0.214
	60-69	274	12.22	26	9.49 (6.56 - 13.54)	*1.71 (0.90 - 3.41)*	0.101	0.105
	70+	262	11.68	15	5.73 (3.5 - 9.23)	*ref*		
	NA	*56*	*2.4*					
**Level of education**	Basic1	160	7.12	11	6.87 (3.88 - 11.89)	*ref*		
	Basic2	129	5.74	10	7.75 (4.26 - 13.68)	*1.14 (0.45 - 2.82)*	0.775	0.775
	Basic3	273	12.14	24	8.79 (5.98 - 12.74)	*1.29 (0.63 - 2.84)*	0.48	0.481
	Medium	276	12.28	26	9.42 (6.51 - 13.44)	*1.4 (0.68 - 3.04)*	0.358	0.36
	Secondary	495	22.02	46	9.29 (7.04 - 12.17)	*1.37 (0.72 - 2.87)*	0.346	0.347
	Superior	845	37.59	87	10.3 (8.42 - 12.53)	*1.54 (0.83 - 3.12)*	0.181	0.184
	No scholarity	70	3.11	7	10.0 (4.92 - 19.23)	*1.51 (0.53 - 4.07)*	0.417	0.419
	NA	*51*	*2.2*					
**Employment status**	Retired	388	17.21	24	6.19 (4.19 - 9.04)	*ref*		
	Employed	1233	54.70	124	10.01 (8.5 - 11.86)	*1.69 (1.09 - 2.72)*	**0.0209**	**0.0222**
	Student	289	12.82	43	14.88 (11.24 - 19.44)	*2.64 (1.57 - 4.53)*	**0.000179**	**0.000272**
	Unemployed	241	10.69	15	6.22 (3.81 - 10.01)	*1.01 (0.51 - 1.96)*	0.984	0.985
	Other	103	4.57	6	5.83 (2.70 - 12.13)	*0.96 (0.34 - 2.28)*	0.892	0.892
	NA	*45*	*2.0*					
**Health profession**	No	1068	89.00	105	9.83 (8.19 - 11.76)	*ref*		
	Yes	132	11.00	13	9.84 (5.85 - 16.12)	*1.01 (0.53 - 1.8)*	0.995	0.995
**Country of birth**	Other countries	414	26.02	39	9.42 (6.97 -12.62)	*ref*		
	Portugal	1177	73.98	126	10.71 (9.06 - 12.60)	*1.15 (0.79 - 1.7)*	0.461	0.461
	NA	*708*	*30.8*					
**Residence locality**	Alcabideche	175	8.48	33	18.86 (13.75 - 25.3)	*ref*		
	Carcavelos / Parede	543	26.32	44	8.10 (6.09 - 10.70)	*0.38 (0.23 - 0.62)*	**6.38E-05**	**0.0001**
	Cascais / Estoril	761	36.89	77	10.12 (8.17 - 12.46)	*0.48 (0.31 - 0.76)*	**1.21E-03**	**0.00145**
	São Domingos de Rana	584	28.31	42	7.19 (5.36 - 9.58)	*0.33 (0.20 - 0.55)*	**5.73E-06**	**1.21E-05**
	NA	*236*	*10.3*					
**Size of househld**	1	222	14.65	17	7.66 (4.84 - 11.92)	*ref*		
	2-3	800	52.81	72	9.0 (7.21 - 11.18)	*1.18 (0.70 - 2.12)*	0.53	0.531
	4-5	431	28.45	63	14.62 (11.59 - 18.26)	*2.05 (1.19 - 3.71)*	0.0102	**0.0115**
	5+	62	4.09	9	14.52 (7.83 - 25.34)	*2.06 (0.83 - 4.82)*	0.0978	**0.103**
	NA	*784*	*34.1*					

Supplementary Table S3 – Multivariable logistic regression using socio-demographic variables as predictors and the result of the serological test (reactive or non-reactive) as outcome, in the first and second periods fo the study. The resulting models are shown for each dataset with the coefficients and p-values of Wald test for each variable, controlling for the other variables.

First period of the study

N=3505; Hosmer and Lemeshow goodness of fit (GOF) test: 0.505

Coefficients:

	Estimate	Std. Error	z value	Pr(>|z|)	
(Intercept)	-4.7269	0.9193	-5.142	0.000000272	***
Sex Male	0.49177	0.27087	1.815	0.0694	.
Age 0-9	3.72488	2.1826	1.707	0.0879	.
Age 10-19	1.73235	1.10372	1.57	0.1165	
Age 20-29	1.45258	0.92643	1.568	0.1169	
Age 30-39	1.72858	0.88576	1.952	0.051	.
Age 40-49	1.21465	0.88126	1.378	0.1681	
Age 50-59	0.72398	0.89467	0.809	0.4184	
Age 60-69	0.74974	0.74482	1.007	0.3141	
Level education Basic2	-0.00708	0.70718	-0.01	0.992	
Level education Basic3	-1.34842	0.78982	-1.707	0.0878	.
Level education Medium	-0.48236	0.67582	-0.714	0.4754	
Level education Secondary	-0.23611	0.60132	-0.393	0.6946	
Level education No scholarity	-1.82127	2.09152	-0.871	0.3839	
Level education Superior	-0.72992	0.61423	-1.188	0.2347	
Employment status Employed	-0.36647	0.65419	-0.56	0.5753	
Employment status Student	-0.88385	0.9442	-0.936	0.3492	
Employment status Unemployed	0.20042	0.69351	0.289	0.7726	
Employment status Other	-0.85661	1.1453	-0.748	0.4545	
Residence locality CARCAVELOS/PAREDE	-0.27365	0.58189	-0.47	0.6382	
Residence locality CASCAIS/ESTORIL	0.6606	0.49913	1.323	0.1857	
Residence locality SÃO DOMINGOS DE RANA	0.31483	0.50887	0.619	0.5361	
Country of birth Portugal	-0.43371	0.29703	-1.46	0.1443	
Size of household 2-3	0.20253	0.3911	0.518	0.6046	
Size of household 4-5	-0.1895	0.44668	-0.424	0.6714	
Size of household 5+	1.41388	0.59804	2.364	0.0181	*

Second period of the study

N=1285; Hosmer and Lemeshow goodness of fit (GOF) test: 0.564

Coefficients:

	Estimate	Std. Error	z value	Pr(>|z|)	
(Intercept)	-1.89262	0.6454	-2.932	0.003363	**
Sex Male	-0.10073	0.19103	-0.527	0.597992	
Age 0-19	-0.62799	1.14227	-0.55	0.582476	
Age 10-19	0.80732	0.77466	1.042	0.297335	
Age 20-29	-0.2998	0.6597	-0.454	0.649503	
Age 30-39	-1.19425	0.7155	-1.669	0.095096	.
Age 40-49	0.09905	0.62104	0.159	0.873283	
Age 50-59	-0.41566	0.61944	-0.671	0.502207	
Age 60-69	0.09763	0.51659	0.189	0.850098	
Level education Basic2	-0.65398	0.66896	-0.978	0.328266	
Level education Basic3	0.03214	0.52969	0.061	0.951619	
Level education Medium	0.20211	0.53833	0.375	0.707327	
Level education Secondary	0.29697	0.50294	0.59	0.554877	
Level education No scholarity	0.70795	0.73398	0.965	0.334773	
Level education Superior	0.60463	0.49856	1.213	0.22523	
Employment status Employed	0.42827	0.48187	0.889	0.374127	
Employment status Student	0.25224	0.66703	0.378	0.705319	
Employment status Unemployed	-0.36641	0.61225	-0.598	0.549536	
Employment status Other inactives	-0.03148	0.73059	-0.043	0.965632	
Residence locality CARCAVELOS/PAREDE	-1.21728	0.31581	-3.855	0.000116	***
Residence locality CASCAIS/ESTORIL	-0.98971	0.29477	-3.358	0.000786	***
Residence locality SÃO DOMINGOS DE RANA	-1.10621	0.30842	-3.587	0.000335	***
Country of birth Portugal	-0.05294	0.22238	-0.238	0.811841	
Size of household 2-3	0.20644	0.32545	0.634	0.525878	
Size of household 4-5	0.54354	0.34525	1.574	0.115406	
Size of household 5+	0.32643	0.51821	0.63	0.528746	

Supplementary Table S4 - Proportion of seropositives for SARS-CoV-2 stratified by chronic diseases, COVID-19 background variables, demand for medical support and absenteeism in the first period of the study. N - sample size for each category; % - proportion of each category within each variable; N sero+ - number of seropositives for SARS-CoV-2; Proportion of seropositives - in percentage and with 95% confidence interval. Association between seropositivity and each categorical variable was analysed with odds ratio, Chi-Square(C-S) test (with continuity correction for tables 2x2) or Fisher (F) exact test (‡), and Wald test from simple logistic regression. Bold values are p-values < 0.05.

First period of the study

Variable	Category	N	%	N sero+	Proportion of seropositives (95% CI) (%)	*Odds ratio (95% CI)*	p-value C-S or F test	p-value Wald test
**At least 1 chronic disease**	No	10889	67.3	155	1.42 (1.22-1.66)	*ref*		
	Yes	5280	32.7	67	1.27 (1.00-1.61)	*0.89 (0.66-1.18)*	0.429	0.429
**At least 2 chronic diseases**	No	14800	91.5	211	1.43 (1.25-1.63)	*ref*		
	Yes	1369	8.5	11	0.80 (0.45-1.43)	*0.57 (0.29-0.99)*	0.058	0.0619
**Diabetes**	No	13964	93.5	203	1.45 (1.27-1.67)	*ref*		
	Yes	966	6.5	8	0.83 (0.42-1.63)	*0.58 (0.26-1.10)*	0.146	0.116
**Chronic lung disease**	No	14321	97	202	1.41 (1.23-1.62)	*ref*		
	Yes	437	3	5	1.14 (0.49-2.65)	*0.83 (0.29-1.83)*	0.795	0.642
**Asthma**	No	13528	90.6	196	1.45 (1.26-1.67)	*ref*		
	Yes	1401	9.4	13	0.93 (0.54-1.58)	*0.64 (0.35-1.09)*	0.144	0.117
**Cardiovascular disease**	No	12664	83.4	181	1.43 (1.24-1.65)	*ref*		
	Yes	2528	16.6	30	1.19 (0.83-1.69)	*0.83 (0.55-1.21)*	0.391	0.342
**Obesity**	No	13694	93.7	191	1.39 (1.21-1.61)	*ref*		
	Yes	921	6.3	16	1.74 (1.07-2.80)	*1.26 (0.72-2.04)*	0.479	0.396
**Autoimmune disease**	No	13313	94.7	198	1.49 (1.30-1.71)	*ref*		
	Yes	746	5.3	9	1.21 (0.64-2.28)	*0.82 (0.39-1.52)*	0.643	0.536
**Kidney disease**	No	3803	98.4	62	1.63 (1.28-2.08)			
	Yes	60	1.5	0	0	-	1 ‡	0.979
**Previous contact with suspected or confirmed case**	No	10281	91.4	81	0.79 (0.63-0.98)	*ref*		
	Yes	967	8.6	99	10.24 (8.48-12.31)	*14.35 (10.62-19.44)*	**1.02E-109**	**<2e-16**
**Did a previous RT-PCR test**	No	13941	83.7	111	0.80 (0.66-0.96)	*ref*		
	Yes	2717	16.3	125	4.60 (3.87-5.45)	*6.01 (4.64-7.79)*	**1.37E-52**	**<2e-16**
**RT-PCR test result**	Negative	2443	96.0	53	2.17 (1.66-2.83)	*ref*		
	Positive	102	4.0	70	68.63 (59.09-76.82)	*97.48 (59.73-161.96)*	**2.39E-203**	**<2e-16**
**Medical support**	No	13322	90.2	125	0.94 (0.79-1.12)	*ref*		
	Yes	1451	9.8	93	6.41 (5.26-7.88)	*7.23 (5.48-9.50)*	**1.54E-60**	
**Absent from work or school**	No	13834	96.3	153	1.11 (0.94-1.29-9	*ref*		
	Yes	532	3.7	55	10.34 (8.03-13.22)	*10.32 (7.43-14.16)*	**1.60E-68**	
**Hospitalized**	No	14493	99	209	1.44 (1.26-1.65)	*ref*		
	Yes	143	1	10	6.99 (3.84-12.39)	*5.21 (2.52-9.57)*	**5.3E-08**	
**Intensive care**	No	4051	99.6	60	1.48 (1.15-1.90)			
	Yes	17	0.4	0	0	-		

Supplementary Table S5 - Proportion of seropositives for SARS-CoV-2 stratified by chronic diseases, COVID-19 background variables, demand for medical support and absenteeism in the second period of the study. N - sample size for each category; % - proportion of each category within each variable; N sero+ - number of seropositives for SARS-CoV-2; Proportion of seropositives - in percentage and with 95% confidence interval. Association between seropositivity and each categorical variable was analysed with odds ratio, Chi-Square(C-S) test (with continuity correction for tables 2x2) or Fisher (F) exact test (‡), and Wald test from simple logistic regression. Bold values are p-values < 0.05.

**Second period of the study**								
**Variable**	**Category**	**N**	**%**	**N sero+**	**Seroprevalence (95% CI) (%)**	***Odds ratio (95% CI)***	**p-value C-S or F test**	**p-value Wald test**
**At least 1 chronic disease**	No	1504	69.5	156	10.37 (8.93 -12.02)	*ref*	*ref*	*ref*
	Yes	660	30.5	47	7.12 (5.40 - 9.34)	*0.66 (0.47 - 0.93)*	**0.017**	**0.0176**
**At least 2 chronic diseases**	No	1999	92.4	194	9.70 (8.48 - 11.08)	*ref*	*ref*	*ref*
	Yes	165	7.6	9	5.45 (2.90 - 10.04)	*0.55 (0.25 - 1.03)*	0.072	0.0763
**Diabetes**	No	1905	93.5	188	9.87 (8.61 - 11.29)	*ref*	*ref*	*ref*
	Yes	133	6.5	6	4.51 (2.08 - 9.49)	*0.44 (0.17 - 0.94)*	**0.042**	**0.0478**
**Chronic lung disease**	No	1987	97.7	187	9.41 (8.21 - 8.20)	*ref*	*ref*	*ref*
	Yes	46	2.3	2	4.35 (1.20 - 14.53)	*0.47 (0.07 - 1.54)*	0.242	0.256
**Asthma**	No	1873	91.4	181	9.66 (8.41 - 11.09)	*ref*	*ref*	*ref*
	Yes	177	8.6	15	8.47 (5.20 - 13.51)	*0.87 (0.48 - 1.47)*	0.607	0.607
**Cardiovascular disease**	No	1757	85.9	170	9.68 (8.38 - 11.15)	*ref*	*ref*	*ref*
	Yes	288	14.1	26	9.03 (6.24 - 12.90)	*0.93 (0.59 - 1.41)*	0.729	0.729
**Obesity**	No	1902	94.4	188	9.88 (8.62 - 11.31)	*ref*	*ref*	*ref*
	Yes	114	5.6	5	4.39 (1.89 - 9.86)	*0.43 (0.15 - 0.97)*	0.053	0.0601
**Autoimmune disease**	No	1884	95.6	185	9.82 (8.56 - 11.25)	*ref*	*ref*	*ref*
	Yes	87	4.4	3	3.45 (1.18 - 9.65)	*0.34 (0.08 - 0.93)*	**0.048**	0.0598
**Kidney disease**	No	1530	98.4	158	10.33 (8.90 - 11.95)	*ref*	*ref*	*ref*
	Yes	25	1.6	1	4.00 (0.21 - 19.54)	*0.41 (0.02 - 1.95)*	0.3	0.321
**Previous contact with suspected or confirmed case**	No	1132	71.5	75	6.63 (5.32 - 8.23)	*ref*	*ref*	*ref*
	Yes	451	28.5	96	21.29 (17.76 - 25.30)	*3.81 (2.75 - 5.28)*	**2.22E-17**	**7.26E-16**
**Did a previous RT-PCR test**	No	1349	61.2	77	5.71 (4.59 - 7.08)	*ref*	*ref*	*ref*
	Yes	855	38.8	136	15.91 (13.61 - 18.51)	*3.12 (2.33 - 4.20)*	**2.88E-15**	**3.13E-14**
**RT-PCR test result**	Negative	742	89.5	56	7.55 (5.86 - 9.67)	*ref*	*ref*	*ref*
	Positive	87	10.5	73	83.91 (74.78 - 90.17)	*62.60 (34.14 - 122.80)*	**3.91E-77**	**<2e-16**
**Medical support**	No	1746	86.2	142	8.13 (6.94-9.51)	*ref*	*ref*	*ref*
	Yes	280	13.8	58	20.71 (16.38-25.84)	*2.95 (2.10-4.12)*	**5.67E-11**	**2.79E-10**
**Absent from work or school**	No	1796	90.7	139	7.74 (6.59-9.07)	*ref*	*ref*	*ref*
	Yes	185	9.3	55	29.73 (23.61-36.67)	*5.04 (3.59-7.21)*	**9.53E-22**	**<2e-16**
**Hospitalized**	No	2006	99.3	200	9.97 (8.73-11.36)	*ref*	*ref*	*ref*
	Yes	15	0.7	1	6.67 (0.34-29.82)	*0.73 (0.03-3.67)*	0.671	0.673
**Intensive care**	No	200	98.5	42	21.0 (15.93-27.17)			
	Yes	3	1.5	0	0	-	-	-
